# Behavioral Patterns in Preschool and School-Aged Children with Snoring and Sleep-Disordered Breathing: A Scoping Review

**DOI:** 10.3390/children12121614

**Published:** 2025-11-27

**Authors:** Diego Monteiro de Carvalho, Carlos Maurício de Almeida, Vinícius Bacelar Ferreira, David Abraham Batista da Hora, Leticia Azevedo Soster, Letícia Rodrigues Nunes Pinheiro, Jefferson Macêdo Dantas

**Affiliations:** 1 Department of Chirurgical Clinic, School of Medicine, University of Amazonas, Manaus 69067-005, AM, Brazil; 2 Department of Neurology, Superior School of Health, State University of Amazonas, Manaus 69850-000, AM, Brazil; 3 School of Medicine, Federal University of Amazonas, Manaus 69067-005, AM, Brazil; davidabrahambdahora@gmail.com; 4 Hospital das Clínicas, Universidade de São Paulo, Sao Paulo 05508-220, SP, Brazil; 5 Hospital Universitário Getúlio Vargas, Federal University of Amazonas, Manaus 69067-005, AM, Brazil; 6 Foundation Hospital Adriano Jorge, State University of Amazonas, Manaus 69850-000, AM, Brazil

**Keywords:** sleep medicine, neurobehavioral manifestations, Sleep Apnea Syndromes, children

## Abstract

**Highlights:**

**What are the main findings?**

• An analysis of 22 recent studies (2019–2024) confirms a strong association between sleep-disordered breathing (SDB) and a broad spectrum of problems, including poor school performance, excessive daytime sleepiness, hyperactivity, and anxiety.
• The central finding is a profound methodological heterogeneity; studies lack standardization in diagnostic tools (despite the common use of polysomnography) and behavioral assessment, which impedes data synthesis.

**What are the implications of the main findings?**

• Clinically, pediatricians and psychologists should maintain a high index of suspicion for SDB in children presenting with unexplained behavioral, emotional, or academic changes.
• For future research, adopting standardized assessment protocols is an urgent necessity to overcome the current heterogeneity, elucidate causal mechanisms, and optimize clinical intervention strategies.

**Abstract:**

**Objective**: This scoping review aims to map the scientific literature of the last five years to characterize behavioral patterns in children with snoring and sleep-disordered breathing (SDB), ranging from primary snoring (PS) to obstructive sleep apnea syndrome (OSAS). The review seeks to identify the main diagnostic and assessment methods, differentiate the behavioral findings between PS and OSAS where possible, and pinpoint key research gaps. **Methods**: A systematic scoping review was conducted following the PRISMA-ScR guidelines. The PubMed/MEDLINE, LILACS, and SciELO databases were searched in April 2024 for observational studies published between 2019 and 2024 that addressed the coexistence of snoring and behavioral changes in the pediatric population. Only articles in English, Spanish, or Portuguese were analyzed. Data were charted and analyzed thematically to map the scope of the evidence. **Results**: The initial search yielded 129 articles, with 22 ultimately included in the final analysis. The findings reveal profound methodological heterogeneity. While questionnaires are universally applied (n = 22), polysomnography (n = 21) remains the gold standard for SDB diagnosis. Behavioral assessments were inconsistent, identifying a broad spectrum of externalizing (e.g., hyperactivity, aggression) and internalizing (e.g., anxiety, depression) problems, with no clear predominant pattern. Obesity and Down’s syndrome were the most frequently associated comorbidities. **Conclusions**: The literature reaffirms the strong correlation between SDB and adverse neurobehavioral outcomes in children. This association is present in primary snoring but is most pronounced in children with diagnosed OSAS. However, progress is constrained by a lack of standardization and, critically, a frequent failure to differentiate between PS and OSAS, which hinders clinical interpretation and evidence synthesis.

## 1. Introduction

Childhood snoring is a prevalent clinical sign that often indicates underlying sleep-disordered breathing (SDB), a spectrum of conditions primarily caused by upper airway obstruction. Among the leading causes are tonsillar and adenoid hypertrophy, which have a prevalence ranging from 42.18% to 70.02% and can lead to varying degrees of complications [1,2]. The resulting intermittent hypoxia and sleep fragmentation are critical pathophysiological mechanisms that appear to be directly related to adverse neurobehavioral outcomes. These consequences often manifest as early as ages 2–6, with potential long-term impacts into adolescence and adulthood [3].

The consequences of SDB on a child’s developing brain can be profound, creating a wide range of behavioral challenges. Clinical evidence links these sleep disturbances to a broad spectrum of consequences. Beyond the widely recognized externalizing behaviors such as inattention and hyperactivity, the clinical picture often includes key physical manifestations like excessive daytime sleepiness (EDS), morning frontal headaches, and secondary nocturnal enuresis, all of which can significantly impair a child’s quality of life and daily routines [1,4]. Furthermore, more severe behavioral disorders, including aggression, bullying, impulsiveness, anxiety, and depression, have been associated with the poorest health indices in this population [5]. These negative behaviors are highly detrimental not only to the children but also to their families, highlighting the urgent need for timely and accurate diagnosis.

Despite the clear association between childhood snoring and behavioral disturbances, the condition remains frequently underdiagnosed. The sheer diversity of neurobehavioral clinical manifestations often leads to misinterpretation of the symptoms, delaying appropriate treatment. This diagnostic challenge is further compounded by the limited availability of polysomnography (PSG), the gold-standard diagnostic tool, for pediatric patients. While snoring is recognized as a cardinal symptom of SDB, such as obstructive sleep apnea (OSA) [6], a comprehensive synthesis that maps the full extent of its associated behavioral phenotypes is currently lacking in the literature.

Given this gap, a scoping review methodology, guided by the PRISMA-ScR framework [7], was chosen as the most appropriate method. This approach is ideal because the topic involves heterogeneous methodologies and diagnostic criteria (e.g., snoring vs. OSAS), and the primary goal is to examine the extent, range, and nature of research activity, identify the key characteristics of the evidence, and, most critically, to summarize findings and identify key gaps in the existing literature [7]. Therefore, the objective of this scoping review is to map the scientific literature of the last five years to characterize behavioral patterns in children with snoring and sleep-disordered breathing (SDB), ranging from primary snoring (PS) to obstructive sleep apnea syndrome (OSAS).

## 2. Methodology

### 2.1. Characterization of the Study

This was a qualitative and quantitative scoping review, focused on children with snoring who develop neurobehavior changes as it is shown in observational studies.

The primary objective was to map and characterize the extent, range, and nature of the scientific literature concerning the behavioral manifestations associated with snoring in the pediatric population.

### 2.2. Data Collection

A systematic search was conducted in the PubMed/MEDLINE, LILACS, and SciELO databases. These databases were chosen to provide comprehensive coverage of both international (PubMed) and Latin American (LILACS, SciELO) biomedical literature, given the review’s multilingual inclusion criteria (English, Spanish, Portuguese).

The search was initially conducted in January, 2024, and the final search was run in April, 2024, and was limited to articles published between 2019 and 2024.

The choice of articles followed the guidelines of the Preferred Reporting Items for Systematic Reviews and Meta-Analyses-PRISMA Extension for Scoping Reviews [7].

The search was conducted in four stages: identification (an initial search covering all articles found using the search words), screening (removal of articles incompatible with the research question by title or abstract), eligibility (application of the inclusion or exclusion criteria), and inclusion (the Stage in which the corpus of the work was established).

Observational studies were used. Studies focusing on specific high-risk populations (e.g., children with Down syndrome) were explicitly included. While these groups present unique neurodevelopmental and physiological confounders, their inclusion is essential to map the full breadth of the SDB literature and to understand its impact in clinically vulnerable populations.

The articles were searched using the following descriptors, in all three bases: ((“Snoring” [Mesh] OR “Sleep Apnea, Obstructive” [Mesh] OR “Sleep Apnea Syndromes” [Mesh]) OR (“Snoring” [tiab] OR “Sleep-Disordered Breathing” [tiab] OR “SDB” [tiab] OR “Obstructive Sleep Apnea” [tiab] OR “OSA” [tiab])) AND ((“Child Behavior Disorders” [Mesh] OR “Neurobehavioral Manifestations” [Mesh] OR “Problem Behavior” [Mesh]) OR (“Behavior” [tiab] OR “Behaviour” [tiab] OR “Behavioral” [tiab] OR “Neurobehavioral” [tiab] OR “Cognitive” [tiab] OR “Attention” [tiab] OR “Hyperactivity” [tiab] OR “Externalizing” [tiab] OR “Internalizing” [tiab] OR “School Performance” [tiab])) AND ((“Child” [Mesh] OR “Adolescent” [Mesh]) OR (“Pediatric” [tiab] OR “Paediatric” [tiab] OR “Child” [tiab] OR “Children” [tiab] OR “Adolescent” [tiab])).

This approach was chosen to build a fundamental conceptual map of literature in which the intersection of these three concepts was the central focus.

After the identification, duplicate articles were removed, and title and abstract screening were performed, eliminating papers that did not address behavioral changes related to sleep disorders or children.

### 2.3. Inclusion and Exclusion Criteria

The inclusion criteria were as follows: (1) Studies must be written in Portuguese, English, or Spanish. (2) Studies must address preschool and school-aged children. For this review, this was operationally defined as an age range including 3 to 18 years, to capture the full pediatric spectrum from preschool entry to high school completion. (3) They must describe the coexistence of sleep disorders and behavioral changes. This was broken down into two distinct operational criteria: (a) Exposure: The study must assess exposure to snoring or SDB. To address the fundamental clinical difference between primary snoring (PS) and obstructive sleep apnea (OSA), we included studies on all forms of SDB, from parent-reported snoring to polysomnography-diagnosed OSA. A primary goal of the data charting was to stratify the included studies based on this distinction (see Table 1). (b) Outcome: The study must assess “behavioral patterns” as an outcome. This was defined as any study that assessed neurobehavioral, cognitive, or emotional outcomes, with a preference for, but not limited to, studies using validated assessment tools (e.g., CBCL, Conners, SDQ, BRIEF). (4) The studies must be observational. (5) Studies must have been published between 2019 and 2024 timeframe. The selection of this recent five-year timeframe was a strategic choice designed to map the state-of-the-art in a rapidly advancing field. This period has been marked by significant progress, including more refined applications of neuroimaging to study brain structure in children with SDB and a deeper exploration of underlying pathophysiological mechanisms. By concentrating on this contemporary period, the review provides a focused synthesis that reflects these modern methodologies and highlights the most pressing questions and definitions currently guiding research and clinical practice.

The exclusion criteria were as follows: (1) articles that did not have an abstract available in the database, (2) articles that addressed the adult population, and (3) studies in which sleep disturbance was secondary to a behavioral disorder.

Using these criteria, papers were selected to establish a corpus of work in the inclusion stage.

### 2.4. Final Selection

Following the final selection, a structured data charting process was undertaken to extract relevant information from the included articles. A data charting form was designed and pre-tested in Microsoft Excel to ensure consistency. Two reviewers independently extracted the data, which included the following: (1) author(s), year of publication (2) study design and sample size; (3) characteristics of the pediatric population (e.g., age range, gender); (4) methods used to assess SDB and behavior; (5) the specific SDB classification used (e.g., Primary Snoring, OSAS, or SDB-unspecified); (6) specific neurobehavioral manifestations reported; and (7) the main findings relevant to the review’s objective.

Concurrently, the methodological quality of each observational study was assessed by the same two reviewers using the Newcastle–Ottawa Scale (NOS) [8]. Any discrepancies in either data extraction or quality assessment were resolved by a third reviewer. It is important to note that the NOS assessment was conducted for descriptive purposes to characterize the state of the evidence; the quality score was not used as a criterion for study exclusion.

Following data extraction, a thematic analysis was conducted by two independent reviewers to map the primary areas of research focus within the included literature. The reviewers identified the principal themes and key terms by systematically comparing the title, abstract, and methodology of each article. This process led to the development of four main categories: (A) Diagnostic Means, (B) Diurnal Changes, (C) Changes in Sleep and Wakefulness, and (D) Associated Pathologies. It is important to note that a single article could be assigned to multiple categories if its content addressed more than one theme.

**Figure 1:**
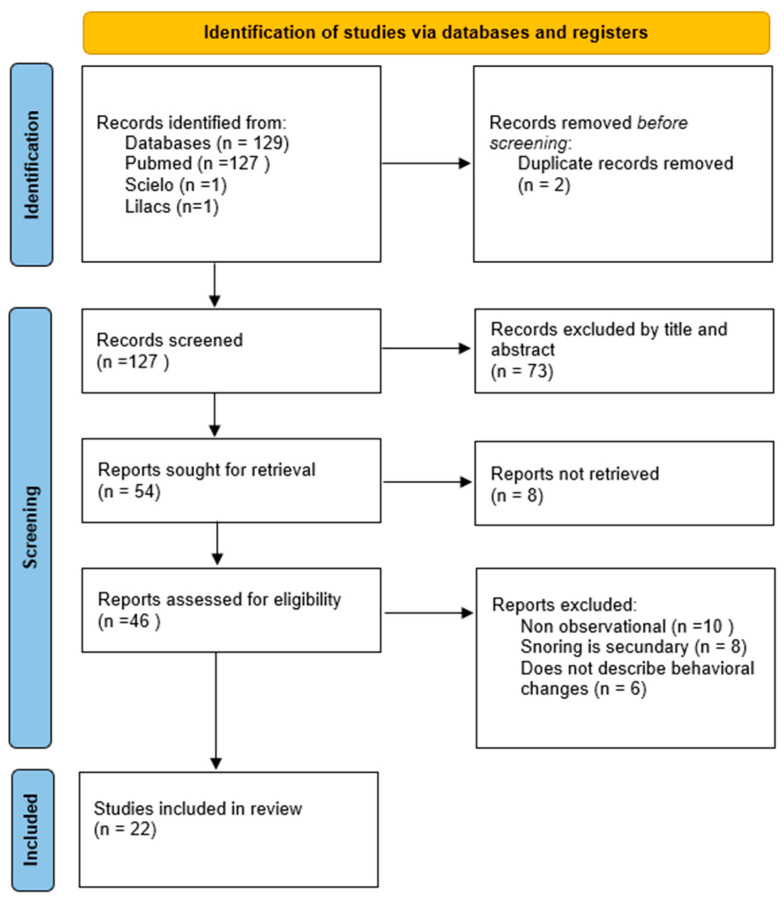
Article selection process. Source: Created by the authors (2025).

The systematic search of the PubMed/MEDLINE, LILACS, and SciELO databases initially identified 129 articles. After the removal of two duplicates, 127 unique articles were screened based on their titles and abstracts. During this screening phase, 73 articles were excluded as they did not address the coexistence of snoring and behavioral changes in children. This left 54 articles for full-text eligibility assessment. Of these, eight articles could not be retrieved, and 24 were excluded for not meeting the inclusion criteria (e.g., being non-observational studies, not describing relevant behavioral changes, or addressing snoring as a secondary outcome). Ultimately, 22 articles fully met the inclusion criteria and were included in the final synthesis. The complete study selection process is detailed in the PRISMA flowchart (Figure 1).

## 3. Results

The 22 included articles were all observational studies published between 2019 and 2024, consistent with the review’s timeframe criteria. A comprehensive summary of each study, including its design, sample size, diagnostic methods, and key findings, is presented in Table 1.

**Table 1:** Main information from the included articles.

Author (Year)	Population and Sample Size (N)	Country	Study Objective	SDB Assessment	SBS Classification	Behavioral Assessment	Key Comorbidities/Focus	Key Behavioral Finding	NOS Score
**Oswald KA (2021)**	Child cancer survivors (N = 75)	USA	To evaluate the prevalence of parent-reported sleep concerns in pediatric cancer survivors and assess the relationship between sleep and neurobehavioral functioning	PSQ	SDB Symptoms	CBCL, TRF	Cancer (Leukemia, Lymphoma)	Increased internalizing and externalizing problems	7
**Shetty M (2023)**	Children with PS and mild OSA (N = 117)	Australia	To examine the effects of SDB on sleep spindle activity in children and its relationship with sleep, behavior, and neurocognition	EEG, PSG	PS and mild OSA	Overall behavioral problems through metabolism/physiology	Primary Snoring, OSA	Lower sleep spindle activity in SDB groups (neurophysiological marker)	5
**Zaffanello M (2023)**	Children with snoring history (N = 47)	Italy	To assess mental health and cognitive development in symptomatic children scoring high on habitual snoring, and the role of obesity and allergy	PSG, Anthropometry	PS and mild OSA	Telephone interviews	Allergies	No significant long-term impact of allergies on quality of life or neuropsychology	8
**Tan B (2023)**	Children with SDB (N = 15)	Australia	To investigate cortical thickness and volumetric changes in children with SDB and how these changes relate to behavioral and cognitive deficits	PSG, MRI	SDB Symptoms	CBCL, BRIEF	SDB	No significant mediating effect of brain structure changes on behavior	9
**Csábi E (2022)**	Children with SDB (N = 78)	Hungary	To assess the behavioral consequences of sleep disturbances by comparing children with SDB (OSA and PS) to a control group	PSG	SDB, OSA, PS	ADHD-RS, SDQ, CBCL	SDB, OSA, PS	SDB group showed more inattention, hyperactivity, internalizing, and externalizing problems	9
**Abou-Khadra MK (2022)**	Preschoolers (N = 319)	Egypt	To determine the prevalence of sleep patterns, problems, and habits in a sample of Egyptian preschoolers	BEARS questionnaire	SDB Symptoms	Overall behavioral problems through metabolism/physiology	General population	Poor sleep hygiene associated with sleep problems (snoring, sleepiness)	6
**Horne RSC (2020)**	Children with SDB vs. controls (N = 533)	Australia	To compare the effects of gender on the severity of SDB, blood pressure, sleep characteristics, quality of life, behavior, and executive function	PSG	SDB Symptoms	Overall behavioral problems through metabolism/physiology	Primary Snoring, OSA	Females with OSA had more internalizing behavioral problems than males	8
**Torres-Lopez LV (2022)**	Overweight/obese children (N = 109)	Spain	To evaluate the associations of parent-reported SDB and device-assessed sleep behaviors with behavioral/emotional functioning in children with overweight/obesity	PSQ (SRBD)	SDB Symptoms	BASC-S2	Overweight/Obesity	SDB associated with attention problems, depression, anxiety, and withdrawal	8
**Hagström K (2019)**	Children with PS vs. controls (N = 831)	Finland	To investigate the neurobehavioral outcomes (behavior, executive functions, QoL) in school-aged children with PSG-diagnosed Primary Snoring	PSG	PS	CBCL, TRF	Primary Snoring	Parents (not teachers) reported more internalizing, total, and attentional problems	6
**Isaiah A (2020)**	Children from ABCD dataset (N = 11,875)	USA	To examine the associations between parent-reported SDB symptoms, behavioral measures (CBCL), and brain morphometry (frontal lobe) in the ABCD dataset	Parent-report (CBCL)	SDB Symptoms	CBCL, Brain Morphometry	SDB, Asthma	Structural changes in gray matter are related to behavioral problems in SDB	9
**Williamson AA (2019)**	Caregiver-child dyads (N = 215)	USA	To examine associations between cumulative socio-demographic risk factors, sleep health habits, and sleep disorder symptoms in young children	PSQ, BCSQ	SDB Symptoms	Overall behavioral problems	Insomnia, OSA	Cumulative risk factors associated with increased OSA symptoms	9
**Siriwardhana LS (2020)**	Children with SDB vs. controls (N = 110)	Australia	To identify the relationship between ventilatory control instability (loop gain) and the severity of SDB in a clinical cohort of children	PSG, Anthropometry	SDB Symptoms	Overall behavioral problems through metabolism/physiology	SDB	Lower GL in children with larger tonsils	8
**DelRosso LM (2021)**	Children with suspected OSA (N = 268)	USA	To define the duration of obstructive apneas and hypopneas in normal children and adolescents to establish normative pediatric data	PSG	OSA	Overall behavioral problems through metabolism/physiology	SDB, OSA	Defined normal apnea/hypopnea duration by age	7
**Liu J (2020)**	Children with snoring (N = 660)	China	To analyze the impact of allergic rhinitis on sleep characteristics and SDB in children with adenotonsillar hypertrophy	PSG, Sleep questionnaire	SDB Symptoms	Sleep questionnaire	Allergic Rhinitis	High prevalence of unspecified behavioral problems in children with allergic rhinitis	6
**Barceló A (2021)**	Children with snoring (N = 137)	Spain	To investigate the inter-relationship between serum 25(OH)D levels (Vitamin D) and parental vitamin D status in a sample of snoring children	PSG	PS	Overall behavioral problems through metabolism/physiology	Hypovitaminosis D	studied link between child and parent Vitamin D levels	9
**Pham TT (2023)**	Patients with Turner Syndrome (N = 151)	USA	To characterize obstructive SDB in young pediatric patients with Turner Syndrome and identify associated risk factors	Clinical diagnosis	SDB	Overall behavioral problems through metabolism/physiology	Turner Syndrome	High prevalence of SDB in Turner Syndrome	6
**Martínez Cuevas E (2021)**	Children with suspected SAHS (N = 67)	Spain	To analyze the association between SAHS and childhood obesity, comparing clinical and polysomnographic characteristics in obese vs. non-obese children	PSG, Anthropometry	OSA	Overall behavioral problems through metabolism/physiology	Obesity	Obese children had less efficient sleep and abnormal metabolism	6
**Walter LM (2019)**	Children with SDB vs. controls (N = 117)	Australia	To determine if SDB in children disrupts the maturation of autonomic control of heart rate and its association with cerebral oxygenation	PSG	SDB	Overall behavioral problems through metabolism/physiology	SDB	Studied autonomic control and cerebral oxygenation in behavior problems	7
**Sivakumar CT (2021)**	Healthy schoolchildren (N = 791)	India	To determine the prevalence of sleep behaviors and their effect on academic performance in schoolchildren aged 6–12 years	CSHQ	SDB Symptoms	CSHQ	General population	High prevalence of altered sleep habits (snoring, parasomnias)	8
**Ezeugwu VE (2022)**	Children with SRBD (N = 165)	Canada	To develop a predictive algorithm (using PSQ and urinary metabolites) to identify pre-school children at risk for behavior changes associated with SDB	PSQ, Actigraphy	SDB Symptoms	Overall behavioral problems through metabolism/physiology	SRBD	Developed predictive algorithm for risk of behavioral problems (unspecified)	7
**McConnell EJ (2020)**	Children with Down Syndrome (N = 120)	UK	To explore the relationship between behavioral and emotional disturbances (using DBC-P24) and SDB symptomatology in a population of children with Down’s syndrome	Epworth Sleepiness Scale	SDB Symptoms	TBPS, PaedESS	Down Syndrome (DS)	SDB symptoms independently associated with worsening behavior	7
**NGO MBH (2024)**	Children with Down Syndrome (N = 44)	Australia	To investigate the relationship between SDB severity in children with Down syndrome and parental psychological wellbeing and social support	PSG	OSA	CBCL, OSA-18	Down Syndrome (DS)	Focused on parental wellbeing, not child’s specific behavioral patterns	6

Abbreviations: ADHD-RS: Attention Deficit Hyperactivity Disorder Rating Scale; BASC-S2: Behavior Assessment System for Children, 2nd ed., Self-Report; BCSQ: Brief Child Sleep Questionnaire; BEARS: Bedtime problems, Excessive daytime sleepiness, Awakenings, Regularity, Snoring; BRIEF: Behavior Rating Inventory of Executive Function; CBCL: Child Behavior Checklist; CSHQ: Children’s Sleep Habits Questionnaire; EEG: Electroencephalography; GL: Glottic Lift; NOS: Newcastle-Ottawa Scale; OSA: Obstructive Sleep Apnea; PaedESS: Paediatric Epworth Sleepiness Scale; PS: Primary Snoring; PSG: Polysomnography; PSQ: Pediatric Sleep Questionnaire; QoL: Quality of Life; SAHS: Sleep Apnea-Hypopnea Syndrome; SDB: Sleep-Disordered Breathing; SDQ: Strengths and Difficulties Questionnaire; SRBD: Sleep-Related Breathing Disorder; TBPS: Total Behavior Problems Score; TRF: Teacher Report Form.

The methodological quality of the studies, as assessed by the Newcastle–Ottawa Scale (NOS), ranged from 5 to 9, indicating a variable level of evidence across the included literature.

The baseline characteristics of the pediatric populations, detailed in Table 1, revealing significant heterogeneity in terms of age, clinical presentation (e.g., primary snoring vs. diagnosed OSA), and the presence of comorbidities.

Data extracted from the 22 articles were thematically categorized to map the primary areas of research focus. The analysis was grouped into four main categories: (A) Diagnostic Means, (B) Diurnal Changes, (C) Changes in Sleep and Wakefulness, and (D) Associated Pathologies.

**Figure 2:**
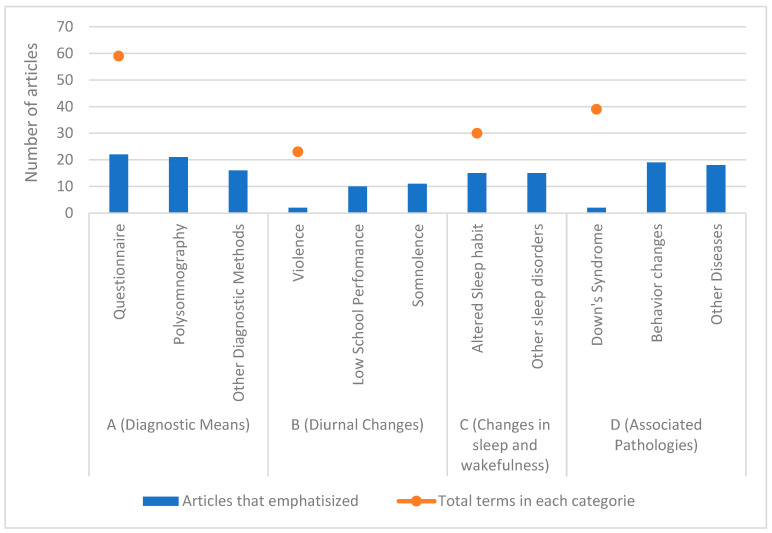
Main elements of each article.

The distribution and frequency of key terms are summarized in Table 2 and visualized in Figure 2. To clarify the quantitative data, the column ‘Total terms in each category’ reflects the sum of articles that mentioned each ‘Principal Term’. For instance, since 22 articles used questionnaires and 21 used polysomnography, both counts contribute to the overall total for the ‘Diagnostic Means’ category, illustrating the prevalence of each specific concept across the literature.

Figure 2 shows the proportion of each category in the terms used, demonstrating the current academic focus in the area.

Source: 2025. Accounting each category, (A) Diagnostic Means: There was a profound heterogeneity in the diagnostic and assessment tools used across the studies. While subjective questionnaires (e.g., PSQ, CBCL, SDQ) were universally applied for behavioral and sleep screening in all 22 studies, polysomnography (PSG) was utilized in 21 of the 22 articles, confirming its status as the gold standard for diagnosing SDB. Other diagnostic methods, including anthropometry, electroencephalography (EEG), and actigraphy, were also employed in 16 studies.

Meanwhile in (B) Diurnal Changes and Behavioral Manifestations: The literature consistently reported a strong association between snoring and a wide spectrum of behavioral problems. Nineteen of the 22 articles described specific behavioral changes. These manifestations included both externalizing problems, such as hyperactivity, aggression, and oppositionality, and internalizing problems, like anxiety, depression, and social withdrawal. However, no single behavioral pattern emerged as predominant. The most frequently reported diurnal consequences were excessive daytime sleepiness (somnolence), noted in 11 articles, and poor school performance, documented in 10 articles.

In (C) Changes in Sleep and Wakefulness: Beyond specific behavioral issues, broader alterations in sleep patterns were a common theme. Fifteen articles highlighted altered sleep habits, and an additional 15 noted the presence of other sleep disorders co-occurring with SDB, such as parasomnias or insomnia symptoms.

At last, (D) Associated Pathologies: Several comorbidities were frequently investigated in relation to snoring and its behavioral consequences. Obesity was a prominent associated condition discussed across literature. Genetic conditions, particularly Down’s syndrome, were also a key area of focus, explored in two dedicated studies. In total, 18 of the articles explored the link between snoring and other co-occurring diseases.

To provide a more granular analysis of the neurobehavioral and clinical manifestations reported in the literature, a systematic inventory of symptoms was compiled from the 22 included articles. The findings were categorized into three primary domains: Behavioral, Cognitive, and Neuropediatric/Clinical. Table 3 details the specific symptoms, their frequency of appearance across the studies, and the corresponding references, offering a quantitative overview of the most reported consequences of sleep-disorder breathing in children.

As detailed in Table 3, the analysis of the included articles reveals a broad spectrum of clinical and neurobehavioral manifestations. The most frequently reported issues were general alterations in sleep patterns, such as parasomnias and poor sleep habits, which were documented in 15 of the 22 articles. Among the diurnal consequences, excessive daytime sleepiness (11 articles) and poor school performance (10 articles) were the most common findings.

In the behavioral domain, externalizing problems, particularly hyperactivity and inattention (8 articles), were slightly more reported than internalizing problems like anxiety and depression (7 articles). It is noteworthy that while a wide range of issues was identified, no single, uniform behavioral phenotype emerged as predominant across literature. These quantitative findings underscore the multifaceted impact of SD.

## 4. Discussion

This scoping review was conducted to systematically map the current evidence regarding the neurobehavioral patterns associated with sleep-disordered breathing (SDB) in preschool and school-aged children, specifically focusing on the distinction between primary snoring (PS) and Obstructive Sleep Apnea Syndrome (OSAS). This comprehensive approach allowed us to identify key characteristics, methodologies, and instruments used across the literature, highlighting existing gaps and informing future research directions.

As such, our scoping review reveals that while the association between sleep-disordered breathing (SDB) and neurobehavioral alterations is unequivocally established in the literature, the field is marked by profound methodological heterogeneity. This variability, particularly in diagnostic criteria and behavioral assessment tools, currently impedes the formation of definitive conclusions regarding causal mechanisms and prevents the establishment of best practices for clinical management. The existing evidence, therefore, provides a broad map of the research landscape but highlights a critical need for standardization to advance our understanding.

Sleep is a complex neurophysiological state whose timing, quality, and architecture change throughout life, and sleep-disordered breathing can profoundly affect neurocognitive development [9]. The volume of research on this topic is substantial. Our analysis of the main terms used (categorized in Table 2 and Figure 2) reveals that the literature is predominantly focused on four key areas: Diagnostic Means, Associated Pathologies, Changes in Sleep and Wakefulness, and Diurnal Changes, in descending order of frequency.

Our mapping of the literature, summarized in Table 3, confirms that SDB is associated with a wide array of neurobehavioral and clinical consequences. To advance the field, it is crucial to move beyond mere association and explore the underlying mechanisms, established knowledge, and unanswered questions for each major symptom domain.

A significant challenge in synthesizing this body of literature is the methodological heterogeneity in diagnosis. The studies reviewed employed a wide range of tools, from subjective assessments like scales and questionnaires to objective tests such as polysomnography (PSG), the latter of which remains the gold standard, but its high cost, limited availability, and the unnatural sleep environment pose significant barriers, especially in pediatrics. This variability in evaluation methods makes it difficult to standardize and compare findings across studies.

Despite these conflicting details, there is a clear consensus across all studies that SDB is associated with significant behavioral alterations. These manifestations, whether externalizing (e.g., aggression, bullying) or internalizing (e.g., anxiety, shyness), negatively impact social interaction, contribute to mental health problems, and lead to poor school performance. Therefore, these behavioral issues should be considered a significant clinical indicator, prompting a thorough investigation for underlying SDB [10,11].

Regarding associated clinical conditions, a comprehensive understanding of SDB requires acknowledging a wide spectrum of comorbidities beyond the frequently cited triad of obesity [12,13,14], allergic rhinitis [12,15], and Down’s syndrome [16,17]. A multitude of pediatric conditions significantly increase the risk for SDB by affecting craniofacial anatomy, neuromuscular tone, or airway patency [9].

Regarding associated clinical conditions, a comprehensive understanding of SDB requires acknowledging a wide spectrum of comorbidities beyond the frequently cited triad of obesity [12,13,14], allergic rhinitis [12,15], and Down’s syndrome [16,17]. A multitude of pediatric conditions significantly increase the risk for SDB by affecting craniofacial anatomy, neuromuscular tone, or airway patency [9]. These include broad categories such as craniofacial syndromes (e.g., Apert, Crouzon), chromosomal and genetic syndromes (e.g., Prader-Willi, Turner Syndrome [18]), neuromuscular disorders (e.g., Duchenne muscular dystrophy), and metabolic storage diseases (e.g., mucopolysaccharidoses), all of which can predispose children to airway obstruction [9]. Recognizing this extensive list of predisposing conditions is clinically vital. It underscores the necessity for targeted SDB screening in these high-risk populations to facilitate early diagnosis and intervention, potentially preventing the severe neurobehavioral sequelae discussed in this review [9]

In populations with DS, the impact of SDB is also a critical area of study. One investigation involving 120 children with DS found that approximately 25% had SDB and that their Total Behavior Problem scores were significantly higher, highlighting the psychopathological burden in this group [16]. Conversely, a different study of 44 children with DS concluded that the severity of the socio-behavioral impact on parents was independent of SDB severity. It also reported an inverse relationship between paternal perception and the emergence of externalizing signs, suggesting a more complex interaction [17]. This divergence, likely influenced by different assessment tools and definitions of ‘severity,’ once again underscores the central challenge of methodological heterogeneity in synthesizing definitive conclusions for specific subpopulations.

Furthermore, evidence suggests that girls with more severe SDB may be more susceptible to developing internalization disorders compared to boys with moderate/severe OSA and to controls [19]. Whether the etiology of this gender-based difference is primarily physiological or psychosocial remains to be elucidated.

Among the diurnal changes, Excessive Daytime Sleepiness (EDS) is one of the most frequently reported symptoms accompanying these behavioral alterations [11,20,21]. EDS directly impairs daily activities requiring attention and concentration, leading to poor school performance and an increased risk of errors and accidents [21]. Inevitably, academic performance is a primary and measurable outcome affected by sleep quality. One study demonstrated that children with the worst school performance not only snored but also exhibited sleep resistance, sleep-related anxiety, and parasomnias like sleepwalking [21]. This finding was corroborated by a study in the Egyptian population, which concluded that both sleep disorders and poor sleep hygiene were directly associated with poor academic performance in preschoolers [22].

A central question emerging from this literature is whether the link between SDB and behavioral disorders is associative or causal. By design, the cross-sectional observational studies mapped in this review can only establish strong patterns of association. They cannot prove SDB causes these changes, as they lack temporal precedence and cannot fully exclude confounders. To bridge this evidence gap, research designs capable of distinguishing causality are required. These include, primarily, prospective longitudinal cohorts to establish that SDB precedes the onset of behavioral deficits, and interventional studies.

It is well-established that the primary mechanisms of SDB—intermittent hypoxia and sleep fragmentation—disrupt key neurodevelopmental processes, particularly in the prefrontal cortex and limbic system. This disruption is a direct cause of the wide spectrum of externalizing (hyperactivity, inattention) and internalizing (anxiety, depression) behaviors observed in these children [10,23].

The biological plausibility for this causal link is strong. The primary pathophysiological mechanisms of SDB—intermittent hypoxia (IH) and sleep fragmentation—are believed to drive these neurobehavioral changes. Recent evidence implicates these mechanisms in triggering cascades of oxidative stress and systemic neuroinflammation, which have a particular impact on the developing brain. These inflammatory pathways are thought to impair the function and maturation of key neural networks, particularly the prefrontal cortex and limbic systems (e.g., anterior cingulate cortex) [10,23]. This disruption provides a direct mechanistic link, as structural alterations in these brain regions have been associated with the spectrum of externalizing (hyperactivity) and internalizing (anxiety) behaviors identified in this review [24,25].

Despite this understanding, critical questions remain. Foremost among them is the source of clinical heterogeneity: Why do children with similar SDB severity present with vastly different behavioral phenotypes with hyperactivity, others with anxiety or predominantly daytime sleepiness? The modulating roles of genetics, sex, and environmental factors are still poorly understood [26,27,28]. Furthermore, the precise dose–response relationship between polysomnographic metrics (e.g., AHI vs. oxygen desaturation levels) and the severity of behavioral outcomes remains elusive. Finally, while it is known that treatment helps, the long-term reversibility of cognitive and behavioral deficits after prolonged SDB exposure is not fully understood [29,30,31].

Considering these findings, our review offers several practical implications. We recommend that pediatricians and child psychologists maintain a high index of suspicion for SDB in children presenting with unexplained academic or behavioral changes. This clinical suspicion should be heightened by the presence of classic physical symptoms, including persistent morning headaches or the new onset of secondary enuresis, as these signs are significant indicators of potential underlying sleep-disordered breathing. Furthermore, the results underscore the urgent need to develop and validate standardized behavioral screening protocols for high-risk pediatric populations, particularly children with obesity, allergic rhinitis, or congenital conditions such as Down syndrome. Such target screening could facilitate earlier diagnosis and intervention, potentially mitigating the long-term neurocognitive and psychiatric consequences of untreated SDB.

In line with the primary goal of a scoping review, our mapping process identified several critical gaps in the literature that must be addressed. First, as noted throughout this review, the field is dominated by heterogeneous methodologies. A significant limitation of the current evidence, as demonstrated in Table 1, is the frequent failure to differentiate between PS and OSA, or the reliance on parent-reported symptoms without objective PSG validation. This makes synthesizing the degree of behavioral impact difficult.

Second, the quality of the evidence (as assessed by the NOS) is variable and largely limited to cross-sectional studies. As discussed, these designs cannot establish causality and are prone to confounding. There is a clear lack of longitudinal studies in the recent literature designed to track neurobehavioral development in children with SDB before and after treatment.

Future research should therefore prioritize: (1) standardized use of diagnostic criteria (PSG) when assessing behavior; (2) prospective, longitudinal cohorts; and (3) interventional studies (e.g., pre- and post-adenotonsillectomy) that use consistent, validated behavioral tools (e.g., CBCL, BRIEF) to measure outcomes.

The conclusions of this review should be considered in the context of its limitations. As a scoping review, our primary objective was to map the extent and range of the available literature. Consequently, we cannot attest to the robustness of each piece of evidence presented, nor can we perform a quantitative synthesis of the data. Secondly, our search strategy was restricted to articles published in English, Portuguese and Spanish, which may have led to the exclusion of relevant research published in other languages. A third significant limitation is our deliberate focus on the 2019–2024 timeframe. While this approach provides a valuable snapshot of the current state-of-the-art, it inherently excludes a vast body of fundamental research published previously that shaped our understanding of SDB.

Despite these constraints, the scoping review methodology was deliberately chosen as the most suitable framework to achieve our primary objective: to comprehensively map the breadth of the evidence, identify key concepts, and pinpoint research gaps in this heterogeneous field, in its modern state. This approach provides a panoramic view necessary to guide the standardization of future research, a task that a more narrowly focused systematic review could not accomplish.

## 5. Conclusions

In conclusion, this scoping review reaffirms the significant link between sleep-disordered breathing and adverse neurobehavioral outcomes in children, ranging from poor academic performance to severe emotional and social challenges. However, the considerable heterogeneity in diagnostic methods and clinical assessments across studies constitutes a major obstacle to progress. While the evidence strongly supports clinical investigation for SDB in children with behavioral concerns, future research must prioritize the adoption of standardized methodologies to clarify causal pathways and optimize intervention strategies, ultimately improving the neurodevelopmental health of affected children.

## Figures and Tables

**Table 2:** Main terms used in the articles.

Terms Categories	Principle Terms	Articles That Emphasized	Total Terms in Each Category
A (Diagnostic Means)	Questionnaire	22	59
	Polysomnography	21	
	Other Diagnostic Methods	16	
B (Diurnal Changes)	Violence	2	23
	Low School Performance	10	
	Somnolence	11	
C (Changes in sleep and wakefulness)	Altered Sleep habit	15	30
	Other sleep disorders	15	
D (Associated Pathologies)	Down’s Syndrome	2	39
	Behavior changes	19	
	Other Diseases	18	

**Table 3:** Clinical Manifestation.

Symptom Domain	Specific Manifestation	Frequency (Nº of Articles)	References (Author, Year)
Behavioral	**Externalizing Problems**	-	-
	Hyperactivity/Inattention	8	Csábi E (2022), Hagström K (2019), Isaiah A (2020), Oswald KA (2021), Siriwardhana LS (2020), Tan B (2023), Torres-Lopez LV (2022), Zaffanello M (2023)
	Aggression/Oppositionality	4	Csábi E (2022), Isaiah A (2020), Oswald KA (2021), Zaffanello M (2023)
	Internalizing Problems	-	-
	Anxiety/Depression	7	Csábi E (2022), Hagström K (2019), Horne RSC (2020), Isaiah A (2020), Oswald KA (2021), Torres-Lopez LV (2022), Zaffanello M (2023)
	Social Withdrawal/Shyness	5	Hagström K (2019), Isaiah A (2020), Oswald KA (2021), Torres-Lopez LV (2022), Zaffanello M (2023)
Cognitive	Poor School Performance	10	Abou-Khadra MK (2022), Csábi E (2022), Ezeugwu VE (2022), Hagström K (2019), Isaiah A (2020), Liu J (2020), McConnell EJ (2020), Shetty M (2023), Sivakumar CT (2021), Zaffanello M (2023)
	Executive Function Deficits	3	Isaiah A (2020), Tan B (2023), Walter LM (2019)
Other Clinical manifestations	Excessive Daytime Sleepiness (Somnolence)	11	Abou-Khadra MK (2022), Csábi E (2022), DelRosso LM (2021), Ezeugwu VE (2022), Horne RSC (2020), Liu J (2020), McConnell EJ (2020), NGO MBH (2024), Pham TT (2023), Sivakumar CT (2021), Zaffanello M (2023)
	Structural Brain Changes	2	Isaiah A (2020), Tan B (2023)
	Altered Sleep Habits/Parasomnias	15	Abou-Khadra MK (2022), Barceló A (2021), Csábi E (2022), DelRosso LM (2021), Ezeugwu VE (2022), Hagström K (2019), Horne RSC (2020), Liu J (2020), Martínez Cuevas E (2021), NGO MBH (2024), Pham TT (2023), Shetty M (2023), Siriwardhana LS (2020), Sivakumar CT (2021), Williamson AA (2019)

## Data Availability

The data presented in this study are available on request from the corresponding author due to privacy.

## References

[1] Brockmann P.E., Gozal D. (2022). Neurocognitive consequences in children with sleep disordered breathing: Who is at risk?. Children.

[2] Pereira L., Monyror J., Almeida F.T., Almeida F.R., Guerra E., Flores-Mir C., Pachêco-Pereira C. (2018). Prevalence of adenoid hypertrophy: A systematic review and meta-analysis. Sleep Med. Rev..

[3] Hiscock H., Davey M.J. (2012). Sleep disorders in infants and children. J. Paediatr. Child Health.

[4] Thabet F., Tabarki B. (2023). Common sleep disorders in Children: Assessment and treatment. Neurosciences.

[5] Csábi E., Gaál V., Hallgató E., Schulcz R.A., Katona G., Benedek P. (2022). Increased Behavioral Problems in Children with sleep-disordered Breathing. Ital. J. Pediatr..

[6] Smith D.L., Gozal D., Hunter S.J., Kheirandish-Gozal L. (2017). Frequency of Snoring, rather than Apnea-hypopnea index, predicts both cognitive and behavioral problems in young children. Sleep Med..

[7] Tricco A.C., Lillie E., Zarin W., O’Brien K.K., Colquhoun H., Levac D., Moher D., Peters M.D.J., Horsley T., Weeks L. (2018). PRISMA Extension for Scoping Reviews (PRISMA-ScR): Checklist and Explanation. Ann. Intern. Med..

[8] Wells G., Shea B., O’Connell D., Peterson J., Welch V., Losos M., Tugwell P. (2021). The Newcastle-Ottawa Scale (NOS) is used to Assess the Quality of Nonrandomized Studies in Meta-Analyses. https://www.ohri.ca/programs/clinical_epidemiology/oxford.asp.

[9] Piotto M., Gambadauro A., Rocchi A., Lelii M., Madini B., Cerrato L., Chironi F., Belhaj Y., Patria M.F. (2023). Pediatric Sleep Respiratory Disorders: A Narrative Review of Epidemiology and Risk Factors. Children.

[10] Walter L.M., Tamanyan K., Weichard A.J., Davey M.J., Nixon G.M., Horne R.S.C. (2019). Sleep-disordered breathing in children disrupts the maturation of autonomic control of heart rate and its association with cerebral oxygenation. J. Physiol..

[11] Hagström K., Saarenpää-Heikkilä O., Himanen S.L., Lampinlampi A.M., Rantanen K. (2019). Neurobehavioral Outcomes in School-Aged Children with Primary Snoring. Arch. Clin. Neuropsychol..

[12] Zaffanello M., Pietrobelli A., Zoccante L., Ferrante G., Tenero L., Piazza M., Ciceri M.L., Nosetti L., Piacentini G. (2023). Mental Health and Cognitive Development in Symptomatic Children and Adolescents Scoring High on Habitual Snoring: Role of Obesity and Allergy. Children.

[13] Torres-Lopez L.V., Cadenas-Sanchez C., Migueles J.H., Henriksson P., Löf M., Ortega F.B. (2022). Associations of Sleep-Related Outcomes with Behavioral and Emotional Functioning in Children with Overweight/Obesity. J Pediatr..

[14] Cuevas E., Peláez C., Carbajo E.O., Eguia A.I.N., Viñe L.M., Jimeno A.P., Alonso-Álvarez M.L. (2021). Sleep apnoea-hypopnoea Syndrome in the Obese and non-obese: Clinical, Polysomnographic and Clinical Characteristics. An. Pediatría.

[15] Liu J., Wu Y., Wu P., Xu Z., Ni X. (2020). Analysis of the Impact of Allergic Rhinitis on Children with Sleep Disordered Breathing. Int. J. Pediatr. Otorhinolaryngol..

[16] McConnell E.J., Hill E.A., Celmiņa M., Kotoulas S.-C., Riha R.L. (2020). Behavioral and Emotional Disturbances Associated with Sleep-disordered Breathing Symptomatology in Children with Down’s Syndrome. J. Intellect. Disabil. Res..

[17] Beng M., Davey M.J., Nixon G.M., Walter L.M., Rosemary S.C. (2024). Horne. Effect of sleep-disordered breathing severity in children with Down syndrome on parental wellbeing and social support. Sleep Med..

[18] Pham T.T., Davis S.M., Tong S., Campa K.A., Friedman N.R., Gitomer S.A. (2023). High Prevalence of Obstructive Sleep-Disordered Breathing in Pediatric Patients With Turner Syndrome. Otolaryngol. Head Neck Surg..

[19] Horne R.S.C., Ong C., Weichard A., Nixon G.M., Davey M.J. (2020). Are there gender differences in the severity and consequences of sleep disorders in children?. Sleep Med..

[20] Oswald K.A., Richard A., Hodges E., Heinrich K.P. (2021). Sleep and Neurobehavioral Functioning in Survivors of Pediatric Cancer. Sleep Med..

[21] Sivakumar C.T., Rajan M., Pasupathy U., Chidambaram S., Baskar N. (2022). Effect of sleep habits on academic performance in schoolchildren age 2021, 6 to 12 years: A cross-sectional observation study. J. Clin. Sleep Med..

[22] Abou-Khadra M.K., Ahmed D., Sadek S.A., Mansour H.H. (2022). Sleep patterns, problems, and Habits in a Sample of Egyptian Preschoolers. Sleep Sci..

[23] Combs D., Goodwin J.L., Quan S.F., Morgan W.J., Parthasarathy S. (2015). Modified STOP-Bang Tool for Stratifying Obstructive Sleep Apnea Risk in Adolescent Children. PLoS ONE.

[24] Isaiah A., Ernst T., Cloak C.C., Clark D.B., Chang L. (2020). Associations between frontal lobe structure, parent-reported obstructive sleep-disordered breathing and childhood behavior in the ABCD dataset. Nat. Commun..

[25] Tan B., Tamanyan K., Walter L.M., Nixon G.M., Davey M.J., Ditchfield M., Horne R.S.C. (2023). Cortical Grey Matter Changes, Behavior and Cognition in Children with Sleep Disordered Breathing. J. Sleep Res..

[26] Shetty M., Perera A., Kadar M., Tan B., Davey M.J., Nixon G.M., Walter L.M., Horne R.S. (2023). The effects of sleep-disordered breathing on sleep spindle activity in children and the relationship with sleep, behavior, and neurocognition. Sleep Med..

[27] Williamson A.A., Mindell J.A. (2019). Cumulative socio-demographic risk factors and sleep outcomes in early childhood. Sleep.

[28] Siriwardhana L.S., Weichard A., Nixon G.M., Davey M.J., Walter L.M., Edwards B.A., Horne R.S. (2020). Role of ventilatory control instability in children with sleep-disordered breathing. Respirology.

[29] DelRosso L.M., Panek D., Redding G., Mogavero M.P., Ruth C., Sheldon N., Blazier H., Strong C., Samson M., Fickenscher A. (2021). Obstructive Apnea and Hypopnea Length in Normal Children and Adolescents. Brain Sci..

[30] Barceló A., Morell-Garcia D., Ribot C., De la Peña M., Peña-Zarza J.A., Alonso-Fernández A., Giménez P., Piérola J. (2021). Vitamin D as a Biomarker of Health in Snoring children: A Familial Aggregation Study. Pediatr. Res..

[31] Ezeugwu V.E., Adamko D., Charmaine van Eeden Dubeau A., Turvey S.E., Moraes T.J., Simons E., Subbarao P., Wishart D.S., Mandhane P.J. (2022). Development of a predictive algorithm to identify pre-school children at risk for behavior changes associated with sleep-related breathing disorders. Sleep Med..

